# Metabolic reprogramming of acute lymphoblastic leukemia cells in response to glucocorticoid treatment

**DOI:** 10.1038/s41419-018-0625-7

**Published:** 2018-08-28

**Authors:** Matheus Dyczynski, Mattias Vesterlund, Ann-Charlotte Björklund, Vasilios Zachariadis, Jerry Janssen, Hector Gallart-Ayala, Evangelia Daskalaki, Craig E. Wheelock, Janne Lehtiö, Dan Grandér, Katja Pokrovskaja Tamm, Roland Nilsson

**Affiliations:** 1 Department of Oncology-Pathology, Cancer Centre Karolinska, Karolinska Institutet, Karolinska University Hospital, 17176 Stockholm, Sweden; 2 Department of Oncology-Pathology, Science for Life Laboratory, Karolinska Institutet, Stockholm, Sweden; 3 Division of Physiological Chemistry 2, Department of Medical Biochemistry and Biophysics, Karolinska Institutet, SE-17177 Stockholm, Sweden; 4 Cardiovascular Medicine Unit, Department of Medicine, Karolinska Institutet, SE-171 76 Stockholm, Sweden; 5 Division of Cardiovascular Medicine, Karolinska University Hospital, SE-171 76 Stockholm, Sweden; 6 Center for Molecular Medicine, Karolinska Institutet, SE-171 76 Stockholm, Sweden

## Abstract

Glucocorticoids (GCs) are metabolic hormones with immunosuppressive effects that have proven effective drugs against childhood acute lymphoblastic leukemia (ALL). Yet, the role of metabolic reprogramming in GC-induced ALL cell death is poorly understood. GCs efficiently block glucose uptake and metabolism in ALL cells, but this does not fully explain the observed induction of autophagy and cell death. Here, we have performed parallel time-course proteomics, metabolomics, and isotope-tracing studies to examine in detail the metabolic effects of GCs on ALL cells. We observed metabolic events associated with growth arrest, autophagy, and catabolism prior to onset of apoptosis: nucleotide de novo synthesis was reduced, while certain nucleobases accumulated; polyamine synthesis was inhibited; and phosphatidylcholine synthesis was induced. GCs suppressed not only glycolysis but also entry of both glucose and glutamine into the TCA cycle. In contrast, expression of glutamine-ammonia ligase (GLUL) and cellular glutamine content was robustly increased by GC treatment, suggesting induction of glutamine synthesis, similar to nutrient-starved muscle. Modulating medium glutamine and dimethyl-α-ketoglutarate (dm-αkg) to favor glutamine synthesis reduced autophagosome content of ALL cells, and dm-αkg also rescued cell viability. These data suggest that glutamine synthesis affects autophagy and possibly onset of cell death in response to GCs, which should be further explored to understand mechanism of action and possible sources of resistance.

## Introduction

Acute lymphoblastic leukemia (ALL) is the most common childhood malignancy, manifested by an expansion of immature B or T cells. Although ALL is genetically heterogeneous, the standard treatment involves the glucocorticoids (GCs) prednisolone and dexamethasone (dex) in combination with other chemotherapeutic agents^1^. While GCs are highly effective treatments, in B-cell precursor ALL (B-ALL) some 20% of patients still relapse and die from the disease, and survivors often suffer lifelong adverse effects due to the treatment^2^. Notably, in vivo and ex vivo GC sensitivity is a good predictor of childhood ALL outcome^3,4^, highlighting the central role of GCs in therapy. Yet, the mechanisms by which GCs kill ALL cells, and the origins of GC resistance, are still unclear. It is known that GC-induced apoptosis depends on GC receptor (NR3C1)-mediated transcriptional induction of its target genes^5–7^. However, GC resistance of ALL in vivo is not simply due to genetic loss of the GC receptor^8,9^, although this frequently occurs in ALL cell lines^10^. A number of GC-regulated mRNAs have been identified^7,11,12^, and gene expression patterns in ALL cells are predictive of GC sensitivity^5,6^, but the underlying molecular mechanisms are not fully understood.

GCs are metabolic hormones that regulate energy metabolism in a variety of tissues in response to hypoglycemia, anoxia, and stresses such as tissue damage^13^. Generally, GCs are catabolic steroids that oppose the action of insulin, inducing a state that resembles insulin resistance. However, distinct cell types respond differently to GCs: in muscle, GCs suppress glucose uptake and glycogen synthesis and cause breakdown of cell protein; while in the liver, GCs induce gluconeogenesis, lipogenesis, and represses fatty-acid oxidation^13^. In addition, GCs can affect cell differentiation and early development, for example, lung development^14^. In various immune cell types, GCs suppress pro-inflammatory signaling and generally inhibit immunological responses^15^.

Despite the known metabolic effects of GCs in other tissues, little is known about the metabolic reprogramming of ALL cells by GCs, and its role in GC-mediated cell death. Several studies have described altered expression of metabolic genes^16–19^, but direct data on metabolite levels or isotope-tracing data, which are essential to demonstrate metabolic activities, are still scarce. GCs cause massive accumulation of autophagosomes in ALL cells^20,21^, indicating a catabolic state similar to nutrient starvation, but the precise metabolic activities associated with this state have, to our knowledge, not been investigated. Like many transformed cells, B-ALL cells exhibit a higher glycolytic rate^22^ than their normal counterparts, and GCs suppress glucose uptake, likely by inhibiting SLC2A1 (GLUT1) expression^23^. However, whether this inhibition of glycolysis is causing cell death, or is a consequence of the cell death program, is not clear. Reducing medium glucose^23^ or treating with 2-deoxyglucose^17,19^ can sensitize B-ALL cells to GCs. Yet, GC-induced immune cell apoptosis is ATP-dependent^24^ and loss of ATP generally leads to necrosis rather than apoptosis^25^, arguing against loss of glycolysis-derived ATP as a mechanism of GC-induced cell death. Moreover, blocking GC-induced autophagy can prevent cell death^20^, indicating that autophagy itself can be detrimental to ALL cells, and that GC-induced cell death is not caused by an energy crisis due to loss of nutrients.

To better understand the metabolic reprogramming caused by GCs, we here report a detailed investigation of the dynamic response of a B-ALL cell line to GC treatment, combining proteomics, metabolomics, and isotope tracing. Consistent with the observed accumulation of autophagosomes, our data indicate a switch from anabolism during cell proliferation to a catabolic state associated with a broad suppression of nutrient uptake. This catabolic state is accompanied by glutamine synthesis, and NH_4_^+^ scavenging by this process may modulate autophagy and possibly cell death.

## Results

### Response of RS4;11 cells to dex

**Fig. 1:**
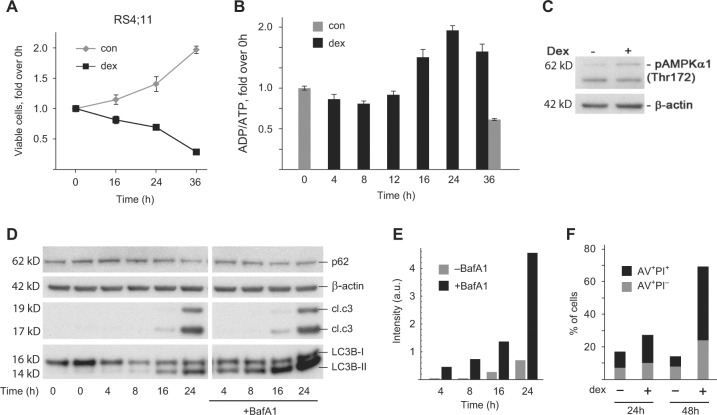
Autophagy and apoptosis in RS4;11 cells in response to the glucocorticoid dexamethasone. **a** RS4;11 cells were either left untreated (con) or treated with 50 nM dexamethasone (dex) for the indicated time points. Number of viable cells were assayed using CellTiterBlue. **b** Cells were cultured and treated as in (**a**) and the ADP/ATP ratio was measured using EazyLight kit. **c** RS4;11 cells were treated as in (**a**) for 24 h and cell lysates were subjected to western blotting with the indicated antibodies. **d** RS4;11 cells were treated as in (**a**) for the indicated time points, and cell lysates were subjected to western blotting with the indicated antibodies. The lysosomal inhibitor bafilomycin A1 (BafA1) was added to the indicated samples for the last 1.5 h of treatments. **e** Quantification of the LC3-II band from (**d**). Images with shorter exposure were used where needed to avoid saturation. **f** Cells were treated as in (**a**) for 24 or 48 h, stained with Annexin V/PI and analyzed by FACS. The fraction of Annexin V+/PI− and Annexin V+/PI+ cells is shown on the same graph to distinguish between early and late apoptotic cells, respectively

The GC-sensitive pre-B ALL RS4;11^26^ cells proliferated in standard culture conditions with a doubling time of ~36 h. Upon treatment with dex, viable cell numbers gradually declined to ~1/3 at 36 h (Fig. 1a). The ADP/ATP ratio was unaffected for the first 12 h, and then increased around 16–24 h (Fig. 1b), indicating a decreased adenylate (energy) charge. Phosphorylation of the AMP-activated protein kinase was also observed at the 24 h time point (Fig. 1c). Autophagosome marker protein LC3-II accumulated between 8 and 16 h, while p62, a protein degraded by autophagy, decreased at 24 h (Fig. 1d). Addition of bafilomycin A1 (BafA1), which blocks lysosomal degradation of autophagosomes, led to a further accumulation of LC3-II and blocked the degradation of p62 at the 24 h time point (Fig. 1d,e), indicating an increased autophagic flux^27^. Cleaved caspase-3 was detectable at 16 h and prominent at 24 h (Fig. 1d), while the fraction of late apoptotic cells (AnnexinV^+^, PI^+^) increased markedly between 24 and 48 h (Fig. 1f). Hence, autophagy precedes onset of apoptosis, similar to our previous report^20^, but continues to increase over time. Thus, GC-treated RS4;11 cells experience a sequence of events including ATP depletion, induction of autophagy, and eventually apoptotic cell death.

**Fig. 2:**
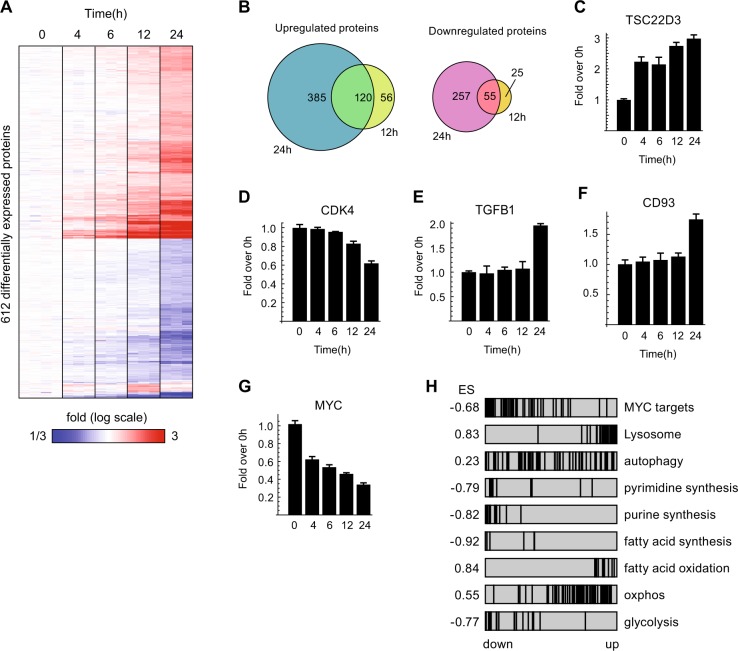
Time-course analysis of the proteome of dex-treated RS4;11 cells. RS4;11 cells were seeded in a custom-made RPMI medium as described in M&M and treated the day after with 50 nM dex. Samples were collected at the indicated time points, proteins were extracted and LC-MS analysis of all proteins was performed as described in M&M. **a** Heatmap of 612 differentially expressed proteins from sample sets in tri-plicate, clustered by Euclidean distance. **b** Overlap of upregulated and downregulated proteins at 24 and 12 h. **c**–**g** Bar charts of selected proteins that were differentially regulated by dex. **h** Enrichment analysis of gene sets at 24 h treatment. Black vertical bars indicate position of individual genes among all genes, sorted by *z*-score. ES denotes enrichment score for each gene set

To characterize the early response to GCs, we performed a high-coverage time-series proteomics analysis of RS4;11 cells following dex treatment, detecting over 7500 proteins (Supplementary Table 1). The resulting data reveal gradually developing changes in protein expression, with the most pronounced differences occurring at 24 h, coinciding with the onset of apoptosis (Fig. 2a,b). Known GC-responsive proteins such as TSC22D3 (Fig. 2c), BCL2L11, FKBP5, SMAP2, TXNIP, and KLF3 (Supplementary Fig. 1A) were all induced in the RS4;11 cells. Proteins involved in cell cycle progression, such as CDK4, decreased over time (Fig. 2d), while antiproliferative proteins such as TGF-beta (TGFB1) increased (Fig. 2e), consistent with cell cycle arrest in leukemic cells. The apoptotic markers BCL2L11 and CD93 emerged at 16–24 h (Supplementary Fig. 1A, Fig. 2f).

The proto-oncogene and transcription factor c-myc (MYC) is known to be downregulated in response to GCs. In our time course data, a decrease of MYC protein were among the earliest events, detectable 4 h after GC exposure (Fig. 2g), was observed on the mRNA level as well (Supplementary Fig. 1B). Moreover, a set of known MYC target genes was significantly downregulated upon dex treatment (Fig. 2h). MYC is known to promote glycolysis in cancer cells through induction of glucose transporters and the glycolytic enzymes hexokinase 2 and lactate dehydrogenase A^28^. Hence, suppression of MYC might be responsible for reduced glycolysis by GCs in leukemia. Taken together, our proteomic data recapitulate known mechanisms and mirror the observed phenotypic changes in RS4;11 cells.

### Metabolic events in GC-treated RS4;11 cells

**Fig. 3:**
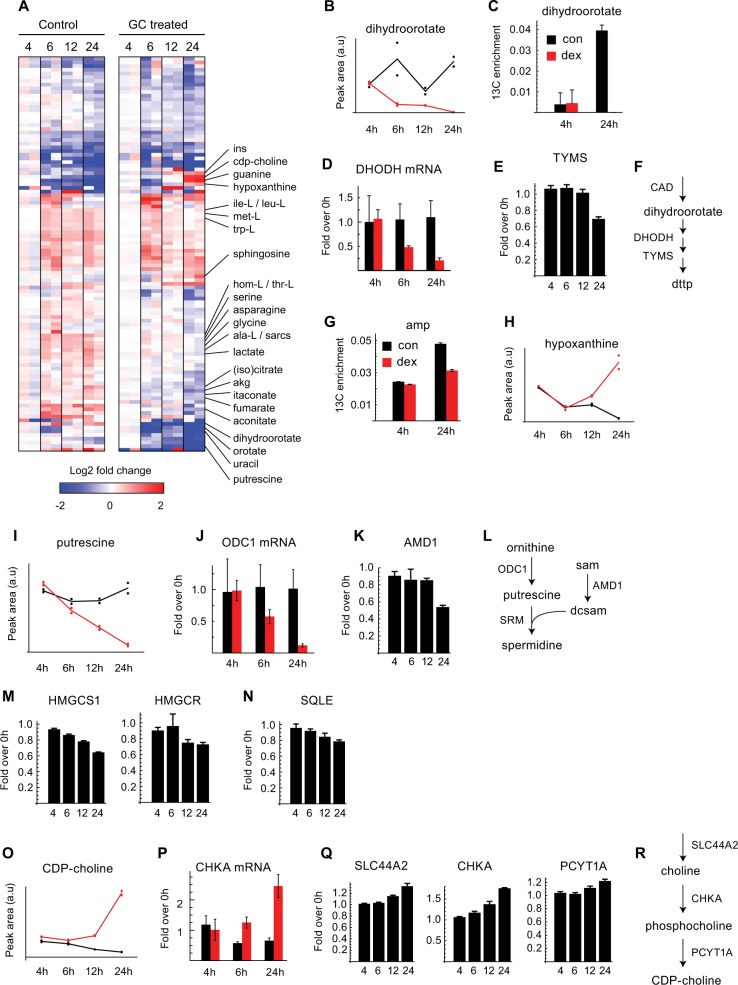
Metabolic profiling and isotope tracing of dex-treated RS4;11 cells. Cells were seeded and treated as in Fig. 2 and metabolomics was performed as described in M&M. **a** Heatmap of relative abundance of metabolites in cell extracts from untreated (left) and GC-treated cells (right) at the indicated time points. Log fold changes over the 4 h time point average are shown. Metabolites of interest are indicated on the right (see text). **b**, **c** Relative abundance (peak areas), and ^13^C enrichment of dihydroorotate, DHODH, mRNA expression measured by RT-PCR (**d**) and TYMS protein measured my LC-MS (**e**) with the schematic view of the pathway in (**f**). **g, h** ^13^C enrichment and relative abundance (peak areas) of adenosine monophosphate (amp) and hypoxanthine, respectively. **i–l** changes in the polyamine synthesis pathway: relative abundance of putrescine (**i**), mRNA expression of ODC1 (**j**), protein levels of AMD1 (**k**), and a schematic of the pathway (**l**). **m**, **n** Protein levels of the mevalonate pathway enzymes HMGCS1 and HMGCR (**m**) and the sterol synthesis enzyme SQLE. **o–r** changes in the CDP-choline pathway: relative abundance of CDP-choline (**o**), CHKA mRNA (**p**), protein levels of the relevant genes (**q**), and schematic of the pathway (**r**). Control (black) and GC-treated cells (red) are labeled uniformly throughout the Figure

To characterize the metabolic effects of GCs over time, we cultured cells in medium containing 1-^13^C-glucose, U-^13^C,^15^N-glutamine, 3-^13^C-serine, and U-^13^C-methionine to trace major metabolic pathways, and analyzed metabolites at several time points after dex treatment and in untreated controls by mass spectrometry (Fig. 3a, Supplementary Table 2). GC treatment caused a rapid decrease in the pyrimidine synthesis intermediates orotate and dihydroorotate (Fig. 3a,b) and prevented ^13^C labeling of dihydroorotate (Fig. 3c), suggesting inhibition of de novo pyrimidine synthesis. Also, mRNA for the pyrimidine synthesis enzyme DHODH declined at 6 h after dex treatment (Fig. 3d,f). In the proteomics data, the pyrimidine synthesis pathway (Fig. 2h) as well as thymidylate synthase (Fig. 3e,f) was downregulated. Similarly, ^13^C labeling of purines (Fig. 3g), and expression of the purine synthesis enzymes (Fig. 2h) declined, consistent with reduced de novo purine synthesis. In contrast, the purine nucleobases hypoxanthine and guanine accumulated in dex-treated cells at 12–24 h (Fig. 3a,h), possibly reflecting nucleotide degradation during apoptosis. We also found a marked decrease in putrescine, the first metabolite of polyamine synthesis (Fig. 3i), as well as decreased mRNA for the putrescine-synthesizing enzyme ornithine decarboxylase (ODC1; Fig. 3j,l) and decreased protein level of adenosylmethionine decarboxylase (AMD1), which supports spermidine synthesis (Fig. 3k,l). Polyamine synthesis is well known to be required by proliferating cells^29^, and ODC1 inhibitors can synergize with GCs to effectively kill ALL cells^30^. Overall, these data demonstrate that GCs cause metabolic effects in ALL cells consistent with suppressed cell proliferation and induced apoptosis.

Although our metabolomics data do not capture sterols or fatty acids, we noted a downregulation of enzymes in fatty-acid synthesis (Fig. 2h), the mevalonate pathway (Fig. 3m), and sterol synthesis (Fig. 3n). Conversely, enzymes involved in fatty-acid oxidation were generally upregulated (Fig. 2h). Previous reports indicate that upregulation of cholesterol synthesis and downregulation of fatty-acid β-oxidation was associated with resistance to GCs in T-cell ALL^16^. Hence, GC-treated cells appear to cease synthesis of fatty acyls and sterols/isoprenoids, and possibly increase oxidation of fatty acids.

Interestingly, CDP-choline, the activated form of choline required for de novo synthesis of most phospholipids, increased markedly at 24 h (Fig. 3o). The choline transporter SLC44A2 was also induced, besides choline kinase (CHKA), and phosphocholine cytidylyltransferase (PCYT1A), which catalyzes synthesis of CDP-choline, as measured by RT-PCR and proteomics (Fig. 3p,q,r). GCs are known to stimulate phosphatidylcholine synthesis in some mammalian cells, for example, in the developing lung^14^, but the role of this process in leukemias is to our knowledge not understood.

### GCs alter fuel usage and promote glutamine synthesis

**Fig. 4:**
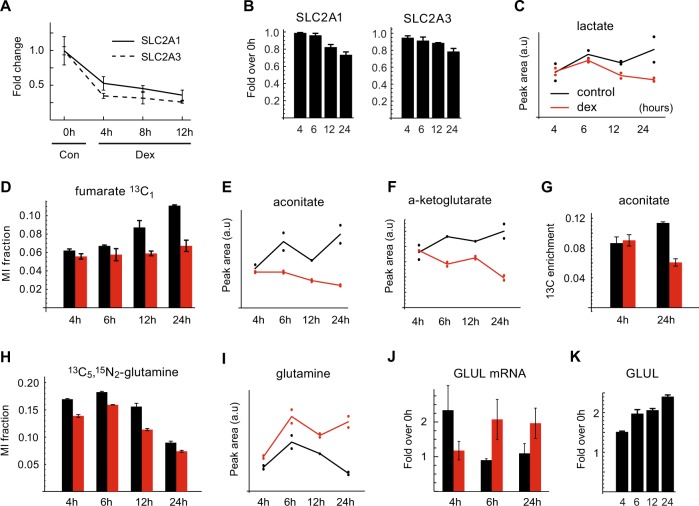
Glucose and glutamine metabolism in dex-treated RS4;11 cells. Measurements of metabolites and enzymes involved in the corresponding pathways in control (con) and dexamethasone (dex)-treated RS4;11 cells at the indicated time points. **a** mRNA levels as measured by RT-PCR and (**b**) protein levels as measured by LC-MS of glucose transporters SLC2A1 and SLC2A3. **c** Relative abundance (peak areas) of lactate (**d–g**) ^13^C enrichment of fumarate (**d**) and aconitate (**g**) and relative abundance of aconitate (**e**) and alpha-ketoglutarate (**f**). **h–i** Fraction of the ^13^C_5_^15^N_2_ mass isotopomer (**h**) and relative abundance (**i**) of glutamine. **j**, **k** mRNA (**j**) and protein (**k**) expression levels of GLUL as measured by RT-PCR and LC-MS, respectively. Control (black) and dex-treated cells (red) are labeled uniformly throughout the Figure

A canonical function of GCs is to inhibit glucose uptake and utilization^13,23^. In RS4;11 cells, glucose transporters SLC2A1 and SLC2A3 were repressed at the mRNA and protein levels (Fig. 4a,b), and lactate accumulated somewhat over time in control cells but not in dex-treated cells (Fig. 4c), consistent with reduced glycolysis. In control cells, the ^13^C_1_ mass isotopomers of TCA cycle metabolites such as fumarate increased over time, likely reflecting entry of ^13^C_1_-acetyl-CoA derived from the ^13^C_1_-glucose tracer, but in dex-treated cells this did not occur (Fig. 4d). This indicates that the contribution of glucose-derived pyruvate to the TCA cycle is diminished in dex-treated cells, which might explain the declining energy charge and AMPK activation (Fig. 1b,c). Yet, treating cells with 2 mM 2-deoxyglucose to inhibit glycolysis^17,23^ did not induce autophagy or cell death to levels comparable to dex treatment (Supplementary Fig. 2A,B), indicating that other mechanisms are important for GC-induced cell death.

Essential amino acids such as leucine, isoleucine, methionine, and tryptophan accumulated over time in untreated but not in GC-treated cells (Fig. 3a), suggesting reduced uptake of these nutrients as well. Measurement of amino-acid concentrations in fresh and spent culture medium confirmed that GC-treated cells have reduced uptake of essential amino acids (Supplementary Fig. 3). TCA cycle metabolites such as aconitate and alpha-ketoglutarate (αKG) were also reduced in dex-treated cells (Fig. 4e,f), and the overall ^13^C enrichment of TCA cycle metabolites decreased over time in dex-treated but not control cells (Fig. 4g), consistent with a switch from oxidation of ^13^C-labeled nutrients (glucose and glutamine) to other, unlabeled fuels, possibly derived from autophagy. Supporting this interpretation, we found a marked increase in lysosomal proteins, in line with the observed induction of autophagy (Fig. 2h). Overall, these data suggest that reduced uptake and utilization of medium nutrients is a general phenomenon of the ALL cell response to GCs.

Since glutamine catabolism can be induced when glucose uptake/metabolism is inhibited^31,32^, one might expect that glutamine uptake would increase in dex-treated cells. However, the fraction of ^13^C_5_^15^N_2_-glutamine (which derives from the glutamine tracer) was lower in dex-treated cells (Fig. 4h), while total glutamine abundance was increased (Fig. 4i). This is consistent with decreased glutamine consumption, and instead suggests increased glutamine synthesis from unlabeled sources. Moreover, the glutamine-synthesizing enzyme glutamate-ammonia ligase (GLUL) was strongly induced by dex treatment at both the mRNA (Fig. 4j) and protein levels (Fig. 4k). Interestingly, this induction of GLUL occurred in GC-sensitive but not in GC-resistant cells in vivo (Supplementary Fig. 4). Neither glutaminase (GLS) nor glutamate dehydrogenase (GLUD) were altered at the protein level upon dex treatment (Supplementary Table 1). Induction of GLUL by corticoids has previously been reported in leukemia cells^33^ and also in normal tissues^34^, but its function in this context remained unclear.

### A possible role for ammonia scavenging by GLUL

**Fig. 5:**
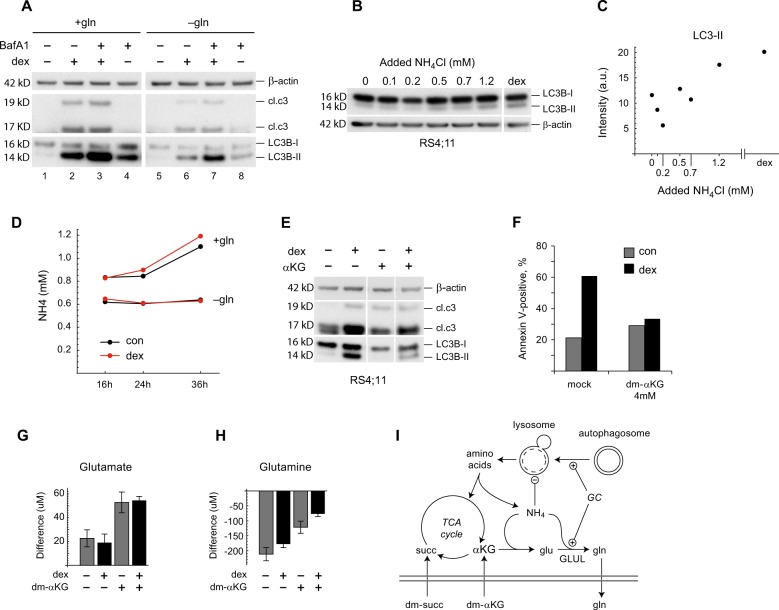
Role of glutamine metabolism in dex-induced autophagy and cell death. **a** RS4;11 cells were seeded either in a complete cell culture medium (+gln) or in the same medium but without glutamine (−gln), left untreated or treated with 50 nM dex for 24 h and indicated proteins’ expression were analyzed using western blotting. BafA1 was added to the indicated samples for the last 1.5 h of treatment to assess autophagic flux. β-actin was used as a loading control. Both panels belong to the same membrane. **b**, **c** RS4;11 cells were seeded as in (**a**), treated with indicated concentrations of NH_4_Cl or 50 nM dex and cell lysates were analyzed for the expression of LC3B (**b**) that was quantified in relation to β-actin (**c**) that was used as a loading control. Both panels belong to the same membrane. **d** RS4;11 cells were seeded and treated as in (**a**) for the indicated time points and ammonia levels in the media were assessed. **e** RS4;11 cells were seeded as in (**a**) and 24 h later treated with either dex alone or in combination with 4 mM of di-methyl-α-KG (KG) for another 24 h and cell lysates were subjected to western blotting with the indicated antibodies. The panels are derived from the same membrane. A representative of two independent experiments is shown. **f** SupB-15 cells were either left untreated or were treated with 50 nM dex for 24 h in the presence or absence of 4 mM dimethyl-α-KG (dm-αKG). Cell death was assessed as percent of all Annexin V+ cells (including Annexin V+/PI+ cells) by FACS. A representative of three independent experiments is shown. **g**, **h** Release of glutamate (**g**) and uptake of glutamine (**h**) to/from the medium. Concentration difference between spent and fresh medium is shown. **i** Schematic of proposed mechanism (see text)

We next asked whether glutamine metabolism might affect autophagy and/or cell death induced by GCs. In untreated cells, removing glutamine from the medium had no effect on LC3-II levels (Fig. 5a, lanes 1 vs. 5), but did reduce autophagic flux measured in the presence of BafA1 (Fig. 5a, lanes 4 vs. 8). On the other hand, in dex-treated cells both LC3-II levels and autophagic flux was clearly inhibited in the absence of glutamine (Fig. 5a, lanes 2 vs. 6, and 3 vs. 7). Also, cleaved caspase 3 was suppressed by lack of glutamine at 24 h (Fig. 5a). Whether glutamine also modulates GC-induced apoptotic cell death was difficult to evaluate, since apoptosis (as measured by Annexin V/PI) in basal conditions was higher in glutamine-free medium (Supplementary Fig. 5), likely because glutamine is required for other metabolic processes^35^. Nevertheless, these data indicate that presence of glutamine modulates autophagy processes.

Because ammonium (NH_4_^+^) is generated by protein catabolism and utilized by the GLUL reaction, and has been reported to inhibit lysosomal degradation^27,36^ and induce autophagy in cancer cell lines^31,32^, we hypothesized that NH_4_^+^ might mediate the observed effects of glutamine on the ALL cell response to GCs. Supplementing medium with increasing amounts of NH_4_^+^ Cl^–^ caused accumulation of autophagy-associated LC3-II, with addition of 1.2 mM being comparable to a 24 h treatment with 50 nM dex (Fig. 5b,c). In glutamine-containing medium, untreated ALL cells produced increasing amounts of NH_4_^+^ over time, while cells in glutamine-free medium did not (Fig. 5d), demonstrating that glutamine accounts for the majority of NH_4_^+^ production in these cultures. NH_4_^+^ production was consistently somewhat higher in dex-treated cells, although the differences were not large; however, intracellular NH_4_^+^ levels may not be fully reflected in spent medium.

A physiological function of the GLUL enzyme is to remove NH_4_^+^ by incorporating it into glutamine, utilizing glutamate that can be derived from α-ketoglutarate (α-kg; Fig. 5i). If dex-induced GLUL does scavenge NH_4_^+^ in ALL cells, then driving the GLUL reaction forward by providing substrate or removing product should relieve the NH_4_^+^-mediated effect on autophagy (Fig. 5i). Remarkably, adding dimethyl-α-ketoglutarate (dm-αkg), which is converted to α-kg in cells^37^, almost completely prevented dex-induced LC3-II accumulation, and also reduced caspase 3 cleavage (Fig. 5e). Removing glutamine had similar effects, as already shown (Fig. 5a). While dm-αkg itself was somewhat toxic for RS4;11 cells during longer treatments, it was well tolerated by Sup-B15 ALL cells for 24 h of treatment, and in this cell line dm-αkg also rescued cell death as monitored by Annexin V/PI staining (Fig. 5f). Addition of dimethyl-succinate, which should lead to an excess of intracellular α-kg, similarly reduced RS4;11 death, as shown previously^23^. Moreover, dm-αkg supplementation increased glutamate production (Fig. 5g) and reduced glutamine uptake (Fig. 5h), consistent with increased synthesis of these metabolites and capture of NH_4_^+^ (Fig. 5i). Taken together, these data suggest that NH_4_^+^ scavenging by the GLUL enzyme is integral to dex-induced catabolism in B-ALL cells, modulating autophagy and possibly cell death in response to GC treatment.

## Discussion

Understanding the mechanisms by which GCs cause cell death is important to find ways to overcome GC resistance, or even substitute GCs with less harmful drugs. In this study, we have described the metabolic reprogramming of ALL cells caused by GC treatment, using a combination of stable isotope tracing, high-resolution mass spectrometry, and large-scale proteomics. Reassuringly, our data recapitulate known effects of GCs, and exhibit metabolic events expected during growth arrest and apoptosis, such as suppression of nucleotide and polyamine synthesis. We also find a coordinated program where fatty acid and sterol synthesis are suppressed, while enzymes in fatty-acid oxidation and oxidative phosphorylation are increased, in line with previous studies^19,38^. While somewhat surprising, this effect might be related to the prominent induction of autophagy, which provides ample material for oxidation^38^. However, increased protein expression does not prove actual increase in oxidative metabolism, and others have reported that respiration is not altered during the first 24 h of GC treatment^39^. Other metabolic phenotypes revealed by this study were previously unknown, including the induction of CDP-choline synthesis, which suggests that phospholipids are synthesized in response to GC treatment. While seemingly counterintuitive, given that GC-treated cells cease biomass synthesis, GCs are known to induce phospholipid synthesis in differentiating cells during lung development^14^, and some studies suggest that membrane synthesis is required by apoptotic cells since “blebbing” increases the surface area of the plasma membrane^40^. Also, formation of autophagosomes requires membrane synthesis; indeed, knockout of a gene involved in CDP-choline synthesis abolished autophagosome formation in fungi^41^.

There are some caveats to the metabolic data presented herein. To capture as many metabolic events as possible, we performed isotope tracing in RS4;11 cells over a 36-h time course, where GCs are administered at time zero. Thus, isotopic transients and changes to metabolic fluxes occur simultaneously, rendering the data more difficult to interpret. For example, an increase in some isotopomer fraction of a metabolite may result from faster synthesis of the metabolite, or reduced pool size (increased turnover), or a change in the corresponding isotopomer fraction of the substrate. Therefore, the metabolic data provided with this study should be considered as suggestive. Also, since GC induces both cell cycle arrest and apoptosis in ALL cells^42,43^, we cannot conclusively attribute specific metabolic events to one or the other without further experiments. However, since apoptosis occurs relatively late in the GC response time course, metabolic effects at earlier time points are more likely to be related to growth arrest. It should also be noted that the metabolic rewiring caused by GCs may be different in vivo, for example, due to hypoxic conditions in the bone marrow where leukemic cells accumulate. Confirmation of our observations in vivo is therefore an important direction for future studies.

The marked induction of the GLUL gene observed in our study has previously been shown in a variety of cell types exposed to GCs^33,34,44^. In muscle, GCs cause a catabolic state with protein breakdown and release of amino acids, of which glutamine is a major component^45^. In this context, GC-induced GLUL acts to capture excess NH_4_^+^ from amino acids into glutamine, which is released into circulation for use by other organs. In our data, several findings point to a similar catabolic phenotype: GCs induce profound autophagy and expression of lysosomal genes, reduce cell amino-acid content and uptake, and suppress entry of both glucose and glutamine into the TCA cycle. These considerations led us to investigate GLUL as a possible ammonia scavenger in ALL cells and involvement of this process in autophagy and cell death.

At high concentrations, NH_4_^+^ can inhibit lysosomal protein degradation^27,36^, indicating a homeostatic mechanism that prevents excessive buildup of NH_4_^+^ from protein catabolism. More recently, NH_4_^+^ at lower concentration ranges have been found to instead stimulate autophagy in carcinoma cells^31,32^, possibly via AMPKβ^46^. In ALL cells, we find that addition of NH_4_^+^ at 1–2 mM result in an accumulation of autophagosomes similar to that observed with GC treatment. It remains to be investigated whether this is due to a downstream block of autophagy^36^ or an increased autophagic flux. Although we could not measure intracellular NH_4_^+^ in GC-treated cells, accumulation of glutamine paralleled by induction of GLUL suggests that GCs induce GLUL-mediated NH_4_^+^ scavenging, coinciding with a massive induction of autophagosomes associated with cell death^20^. Addition of dm-αkg or removal of glutamine from the medium inhibited autophagy and cell death, and dm-αkg altered metabolism in a manner consistent with increased NH_4_^+^ assimilation by GLUL, leading us to a hypothetical model where GLUL impacts autophagy via NH_4_^+^, which in turn modulates apoptosis (Fig. 5i). It should be mentioned that the role of autophagy in relation to cell death has been controversial^20,21,38^. We have previously found that the profound autophagy induced by dex in pre-B-ALL cell lines contributed to apoptosis^20^, while other studies instead suggest a cytoprotective role^21,38^. A simple reason for this discrepancy could be that our previous^20,23,47^ and present work concerns B-ALL cells, which differ from the mouse and human T-ALL experimental models of other studies^21,38^. Also, chloroquine, which inhibits autophagy at the lysosome fusion step, potentiated dex-induced apoptosis^38^, while we have observed that inhibition of early autophagosome formation prevents apoptosis^20^. Clearly, additional studies are required to fully understand the different effects of these interventions.

Interestingly, low GLUL expression is predictive of poor outcome and relapse in ALL^16,48^, supporting the notion that GLUL is an integral, functional part of the GC response. In line with this, in GC-treated primary ALL mouse xenografts^12^, induction of GLUL by dex occurred in GC-sensitive but not in GC-resistant primary cells. For these reasons, we believe the GLUL enzyme merits further examination to better understand the role of autophagy and catabolism in GC-induced ALL cell death.

## Materials and Methods

### Cell lines, culture conditions, and treatments

Two pre-B ALL cell lines, RS4;11 (ATCC, no. CRL-1873, USA) and SupB-15 (DSMZ, no. ACC 389, Germany), were used in this study. The cells were cultured in RPMI 1640 medium (Gibco no. 22400089) containing 25 mM HEPES for the RS4 and SupB15 cell lines, supplemented with 10% (v/v) heat-inactivated fetal calf serum (Gibco no. 10270106), 2 mM L-glutamine (Gibco no. 25030-024), 50 μg/ml streptomycin and penicillin (Gibco no. 15140122) and maintained in a humidified incubator in 5% CO_2_ at 37 °C. For the experiments, cells were seeded at 400,000 cells/ml and treated the day after as indicated for each experiment. For treatments of cells, the following reagents used were all from Sigma-Aldrich Chemie GmbH: dex (no. D1756) at a variety of concentrations indicated in each experiment, 2-deoxy-glucose (no. D6134-G) at final concentration of 2 mM, methyl pyruvate (no. 371173) at final concentration of 2 mM, N-acetyl-D-glucosamine (GA, no. A3286) at final concentration of 35 mM, αKG (no. 349631) at final concentration of 4 mM, were used for single treatment as well as 1 h pretreatment before Dex addition. Ammonium chloride, NH_4_Cl (Merck no. 12125-02-9) was used at 1–2 mM in sterile water. BafA1 (Selleck chemicals no. S1413) was used at 10 nM concentration for the last 1.5 h of dex or NH_4_Cl treatment. For the measurement of ammonia in cell media, the Ammonia assay kit was used (BioVision #K370) according to the manufacturer’s instructions. The standard curve was made using NH_4_Cl solutions for each experiment. ADP/ATP ratio was determined using EazyLight ADP/ATP assay kit (BioAssay Systems no. ELDT-100) according to the manufacturer’s instructions.

### Metabolomics and isotope tracing

For the metabolomics and isotope-tracing experiments, a custom-synthesized RPMI medium was used according to standard formulation (Sigma), using the following isotopomers: 70% 1-^13^C-glucose, 50% U-^13^C, ^15^N-glutamine, 60% 3-^13^C-serine, and 50% U-^13^C-methionine. For metabolite extraction, cells were washed twice with Hank’s balanced salt solution, and the resulting pellet was extracted with cold HPLC-grade methanol and analyzed using a Thermo QExactive orbitrap instrument coupled to a HILIC chromatography system, as previously described^49^. The identity of metabolites were confirmed by matching retention times against those of pure standards, and additionally supported by comparing the observed mass isotopomers to those expected given the isotope tracers used. We identified 107 metabolites in this manner, and for each integrated peaks for all possible carbon and nitrogen isotopomers, comprising a total of 2720 mass isotopomers. Relative abundance for each metabolite was calculated as the sum of all mass isotopomer peak areas. To account for instrument drift, relative abundance data were normalized to the median of all metabolites in each sample. The full data set is available in Supplementary Table 2.

### Quantitation of medium metabolites

Supernatants from cell culture (spent medium) or fresh medium incubated without cells in the same conditions (fresh medium) were used for quantification, mixed 1:1 with a water solution of quantitative ^13^C standards for the metabolites measured. Samples were vortexed for 15 s, centrifuged for 2 mins at 19,000×*g* to remove debris, mixed 1:4 with methanol, vortexed for 30 s, incubated at room temperature for 10 min, and finally centrifuged for 15 min at 12,000×*g*. LC-HRMS experiments were performed on a 1290 Infinity II ultrahigh performance liquid chromatography (UHPLC) system coupled to a 6550 iFunnel quadrupole-time of flight (Q-TOF) mass spectrometer equipped with a dual AJS electrospray ionization source (Agilent Technologies, Santa Clara, CA, USA). Polar metabolites were separated on a SeQuant® ZIC®-HILIC (Merck, Darmstadt, Germany) column 100 Å (100 mm × 2.1 mm, 3.5 µm particle size) coupled to a guard column (20 mm × 2.1 mm, 3.5 µm particle size) and an inline filter. Mobile phases consisted of 0.1% formic acid in water (solvent A) and 0.1% formic acid in acetonitrile (ACN; solvent B). The elution gradient used was as follows: isocratic step at 95% B for 1.5 min, 95% B to 40% B in 12 min, and maintained at 40% B for 2 min, then decreasing to 25% B at 14.2 min and maintained for 2.8 min, and then returned to initial conditions over 1 min, and the column was equilibrated at initial conditions for 7 min. The flow rate was 0.3 mL min^−1^, injection volume was 2 µL and the column oven was maintained at 25 °C. Two independent injections were run for positive and negative acquisition modes. The Q-TOF MS system was calibrated and tuned according to the protocols recommended by the manufacturer. Nitrogen (purity > 99.9990%) was used as a sheath gas and drying gas at a flow of 8 and 15 L min^−1^, respectively. The drying and sheath gas temperature were set at 250 °C, with the nebulizer pressure at 35 psi and voltage 3000 V (+/− for positive and negative ionization modes, respectively). Full-scan high-resolution data were acquired with a mass range of 40–1200 *m*/*z*. The data acquisition rate was 6 scans s^−1^. LC-MS grade water and formic acid (Optima®-LC/MS) were purchased from Sigma-Aldrich (St. Louis, USA). ACN (Optima®-LC/MS) and methanol (Optima®-LC/MS) were purchased from Fisher-Scientific (Loughborough, UK). For the LC-HRMS experiments, the internal lock masses (purine and HP-0921) and tune mix for calibrating the Q-TOF-MS (ESI-low concentration tuning mix) were purchased from Agilent Technologies (Santa Clara, USA). Medium metabolite concentrations were calculated from mass isotopomers' peak ratios, given the known concentration of each ^13^C-labeled standard.

### Viability, apoptosis, and autophagy detection

For the viability assays, CellTiterBlue (Promega no. G8081) was used according to the manufacturer's instructions and each treatment was done in triplicate. When medium without glutamine was used, a WST-1 viability assay was used instead (Roche/Sigma no. CELLPRO-RO). For Annexin V/propidium iodide (PI) stainings and fluorescence-activated cell sorting (FACS) analysis, 4 × 105 – 1 × 106 cells were collected and washed with PBS. Cells were incubated for 20 min with AnnexinV and PI stains (BD Pharmingen no. 556463) in Annexin V buffer (10 mM HEPES, 140 mM NaCl, 5 mM CaCl_2_) at +4 °C. Stained cells were analyzed for apoptosis and cell death by FACS Calibur flow cytometer (BD) using Cell Quest software (BD). Cells were gated to include only single cells; 10,000 cells were counted in this gate for each sample. Annexin V positivity marks apoptotic cells, whereas double Annexin V/PI positivity marks later stage in apoptosis. Typically, all Annexin V-positive cells were taken into analysis and presented in the bar charts unless otherwise indicated.

### RNA extraction and cDNA

RNA was extracted by using the NucleoSpin® RNA kit (Macherey-Nagel no. 740955.250) following the manufacturer's instruction and treated with DNase (Ambion Turbo DNA-free, Life Technologies). RNA (~500 ng) was used for the generation of cDNAs by using MuMLV Reverse Transcriptase (Life Technologies no. 28025013) and a mixture of 50% oligo(dT)18 and 50% random nanomers following the manufacturer's manual.

### qRT-PCR

qRT-PCR quantification was carried out by using the KAPA 2 G SYBRGreen (Kapa Biosystems) on the Applied Biosystems 7900HT platform with the following cycling conditions: 95 °C for 3 min, 95 °C for 3 s, 60 °C for 30 s. The primers used were (gene symbol, forward primer, and reverse primer): GLUL, aagagttgcctgagtggaatttc, agcttgttagggtccttacgg; DHODH, ggaaaccctagacccagagtc, accactgaaagcccgtgac; ODC1, gccatcgtgaagacccttg, ggcaatccgcaaaaccaactt; CHKA, gaaatcgccgagaaaatggct, gggcagattgtaactgagcaa; CMYC, ggtagtggaaaaccagcagc, tcctcgtcgcagtagaaatac; SLC2A1, catcaatgccccccagaa, aagcggcccaggatcag; SLC2A3, gggcatcgttgttggaattctggt, agggctgcactttgtaggatagca; ACTB, aggtcatcaccattggcaatgag, ctttgcggatgtccacgtca.

### Sample preparation for mass spectrometry-based proteomics

The cell pellets were lysed by addition of lysis buffer: 4% SDS, 1 mm DTT, and 25 mm HEPES pH 7.6, followed by heating to 95 °C for 5 and 1 min sonication of the lysate. Protein concentration was determined by Bio-Rad DCC protein assay. Samples were digested by a modified FASP protocol^50^. Briefly, 250 µg of each sample was mixed with 1 mM DTT, 8 M urea, 25 mM HEPES, pH 7.6 and transferred to a 10-kDa cutoff centrifugation filtering unit (Pall, Nanosep®), and centrifuged at 14,000×*g* for 15 min. Proteins were alkylated by 50 mM iodoacetamide in 8 M urea, 25 mM HEPES for 10 min. The proteins were then centrifuged at 14,000×*g* for 15 min followed by two more additions and centrifugations with 8 M urea, 25 mM HEPES. Proteins were digested at 37 °C with gentle shaking overnight by addition of trypsin (enzyme:protein = 1:50, Thermo Fisher Scientific) in 250 mM Urea, 50 mM HEPES pH 7.6. The filter units were centrifuged at 14,000×*g* for 15 min followed by another centrifugation with MQ and the flow-through was collected. Peptide concentration was determined by the Bio-Rad DCC assay and 100 µg of peptides from each digested fraction was labeled with TMT10plex reagent according to the manufacturer’s protocol (Thermo Scientific). Labeled samples were pooled, cleaned by strata-X-C-cartridges (Phenomenex), and dried in a Speed-Vac.

### Peptide-level sample fractionation through IPG-IEF

The TMT-labeled peptides, 300 µg, were separated by immobilized pH gradient-isoelectric focusing (IPG-IEF) on pH 3–10 strips as described^50^. Peptides were extracted from the strips by a prototype liquid-handling robot, supplied by GE Healthcare Bio-Sciences AB. A plastic device with 72 wells was put onto each strip and 50 µl of MQ was added to each well. After 30 min incubation, the liquid was transferred to a 96-well plate and the extraction was repeated two more times with 35% ACN and 35% ACN, 0.1% formic acid in MQ, respectively. The extracted peptides were dried in Speed-Vac and dissolved in 3% ACN and 0.1 % formic acid.

### Mass spectrometry-based quantitative proteomics

Extracted peptide fractions were separated using an Ultimate 3000 RSLCnano system coupled to a Q Exactive or Q Exactive HF (Thermo Fischer Scientific, San Jose, CA, USA). Samples were trapped on an Acclaim PepMap nanotrap column (C18, 3 µm, 100 Å, 75 µm × 20 mm), and separated on an Acclaim PepMap RSLC column (C18, 2 µm, 100 Å, 75 µm × 50 cm; Thermo Scientific). Peptides were separated using a gradient of A (5% DMSO and 0.1% FA) and B (90% ACN, 5% DMSO, and 0.1% FA), ranging from 6 to 37% B in 30–90 min (depending on IPG-IEF fraction complexity) with a flow of 0.25 µL min^−1^. The Q Exactive was operated in a data-dependent manner, selecting top 10 precursors for fragmentation by HCD. The survey scan was performed at a resolution of 70,000 (FWHM) from 400 to 1600 *m*/*z*, with a max injection time of 100 ms and target of 1 × 10^6^ ions. For generation of HCD fragmentation spectra, a max ion injection time of 140 ms and AGC of 1 × 10^5^ were used before fragmentation at 30% normalized collision energy at 35,000 resolution (FWHM). Precursors were isolated with a width of 2 *m*/*z* and put on the exclusion list for 70 s. Single and unassigned charge states were rejected from precursor selection.

### Peptide and protein identification

Orbitrap raw MS/MS files were converted to mzML format using msConvert from the ProteoWizard tool suite^51^. Spectra were then searched using MSGF+ (v10072^52^) and Percolator (v2.08^53^), where search results from eight subsequent fraction were grouped for Percolator target/decoy analysis. All searches were done against the human protein subset of Ensembl 75 in the Galaxy platform^54^. MSGF+ settings included precursor mass tolerance of 10 ppm, fully tryptic peptides, maximum peptide length of 50 amino acids, and a maximum charge of 6. Fixed modifications were TMT-10plex on lysines and peptide N termini, and carbamidomethylation on cysteine residues, a variable modification, was used for oxidation on methionine residues. Quantification of TMT-10plex reporter ions was done using OpenMS project’s IsobaricAnalyzer (v2.0^55^). PSMs found at 1% false discovery rate (FDR) were used to infer gene identities.

Protein quantification by TMT10plex reporter ions was calculated using TMT PSM ratios to the entire sample set (all 10 TMT channels) and normalized to the sample median. The median PSM TMT reporter ratio from peptides unique to a gene symbol was used for quantification. Protein FDRs were calculated using the picked-FDR method using gene symbols as protein groups and limited to 1% FDR^56^.

### Statistical analysis and data analysis

For the heatmap of proteomics data (Fig. 2a), genes were included that differed from the 0 h time point with a *z*-score of at least four and a fold change of at least 1.3 at any time point; a total of 612 genes passed this criterion. Proteins were ordered using hierarchical clustering with the Euclidean distance metric and average linkage. The sets of differentially expressed genes at the 12 and 24 h time points (Fig. 2b) were defined as significantly upregulated or downregulated genes at the given time point as compared to untreated cells, with a fold change outside of the 99% confidence interval for the untreated replicates (~>1.2 or ~<0.8). The ES was calculated using the GSEA-p method^57^ with *p* = 1.

## Electronic supplementary material

Supplementary Figure legends

Supplementary Figure 1

Supplementary Figure 2

Supplementary Figure 3

Supplementary Figure 4

Supplementary Figure 5

Supplementary Table 1

Supplementary Table 2
