# Supplementary material for: Metabolic reprogramming of acute lymphoblastic leukemia cells in response to glucocorticoid treatment
Source: Cell Death Dis. 2018 Aug 28;9(9):846. doi: 10.1038/s41419-018-0625-7 (PMC6113325; doi:10.1038/s41419-018-0625-7)
Supplement: Supplementary file 1 — Supplementary Figure legends [file 41419_2018_625_MOESM1_ESM.docx]

**Supplementary figure legends**

**Supplementary Figure 1**. (A) The data from LC-MS analysis of RS4;11 cells, as described in Fig. 2. Bar charts of protein expression of a panel of known GC-regulated genes at the indicated time points after dex treatment as compared to Ctrl untreated cells. (B) RS4;11 or Sup-B15 ALL cells were seeded and treated the day after with 50 nM dex for the indicated time points and relative c-myc mRNA expression was measured by RT-PCR. β-actin gene expression was used as control. Ranked Gene set enrichment analysis of changes at 24 h treatment.

**Supplementary Figure 2.** (A) RS4;11 cells were seeded and treated the day after with either 75 nM dex (Lane 2 and 6) or 2 mM 2DG (lanes 3 and 7), or 1,2 mM NH4Cl (lanes 4 and 8). 10 nM BafA1 was added, as indicated, in the last 1,5 h of treatments. Cell lysates were subjected to Western blotting with the indicated antibodies. (B) RS4;11 cells were seeded and the day after treated with either 50 nM dex or 2 mM 2DG for 36 h. Cell death was assessed by Annexin V/PI staining and FACS analysis. The amount of all Annexin V-positive cells (including Annexin V+/PI+ cells) is presented as bar charts from two independent experiments.

**Supplementary Figure 3.** Uptake of essential amino acids in the conditions indicated. Concentration difference between spent and fresh medium is shown.

**Supplementary Figure 4.** GLUL expression after in vivo dexamethasone treatment (red) and controls (grey) of xenotransplanted primary ALL cells from glucocorticoid resistant (R) (DEXA vs control p=0.8788, paired t-test) and sensitive (S) patients (p=0.0057), each with 2-3 replicates per leukemia sample. Original data from Jing et al [12] available via GEO accession GSE57795.

**Supplementary Figure 5.** RS4;11 cells were seeded and treated as in Fig. 5A, stained with Annexin V/PI and analyzed by FACS. Summary of three independent experiments is shown. Cell death was assessed as per cent of all Annexin V+ cells including Annexin V+/PI+ cells.
