# Supplementary figures and images for: Metabolic reprogramming of acute lymphoblastic leukemia cells in response to glucocorticoid treatment
Source: Cell Death Dis. 2018 Aug 28;9(9):846. doi: 10.1038/s41419-018-0625-7 (PMC6113325; doi:10.1038/s41419-018-0625-7)

Supplementary Figure 1

A

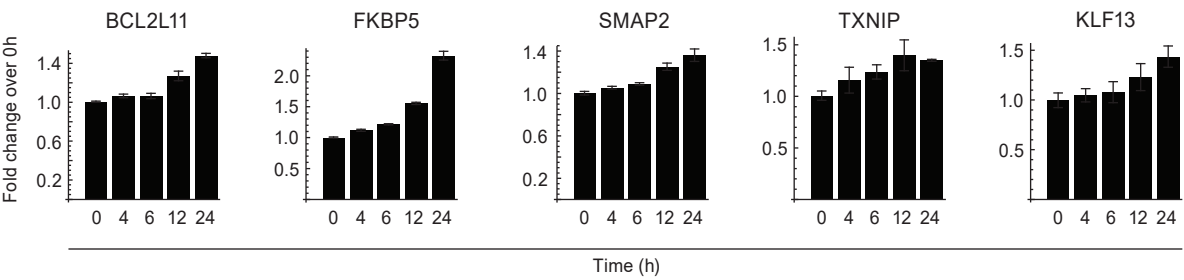

B

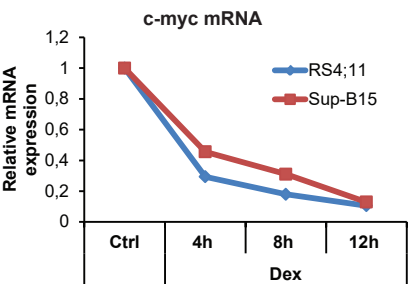

Supplement: Supplementary file 2 — Supplementary Figure 1 [file 41419_2018_625_MOESM2_ESM.pdf]

Supplementary Figure 2

A

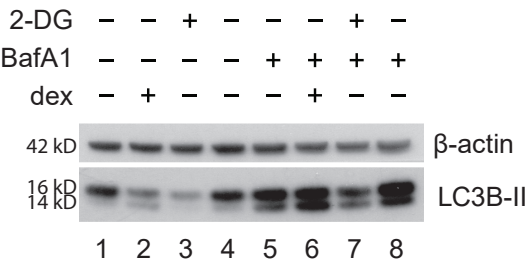

B

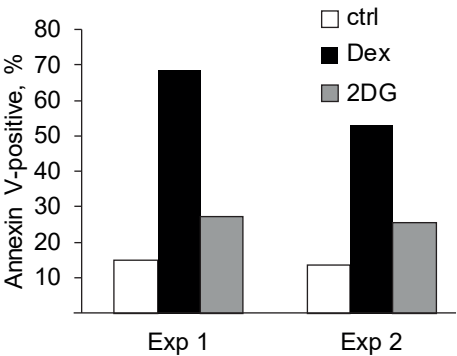

Supplement: Supplementary file 3 — Supplementary Figure 2 [file 41419_2018_625_MOESM3_ESM.pdf]

Supplementary Figure 3

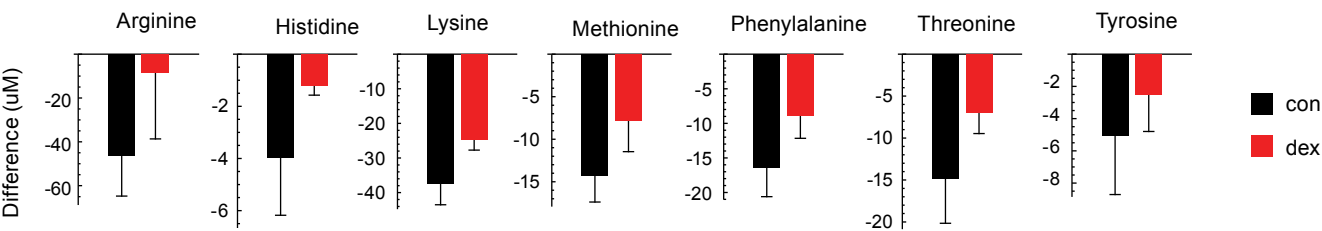

Supplement: Supplementary file 4 — Supplementary Figure 3 [file 41419_2018_625_MOESM4_ESM.pdf]

Supplementary Figure 4

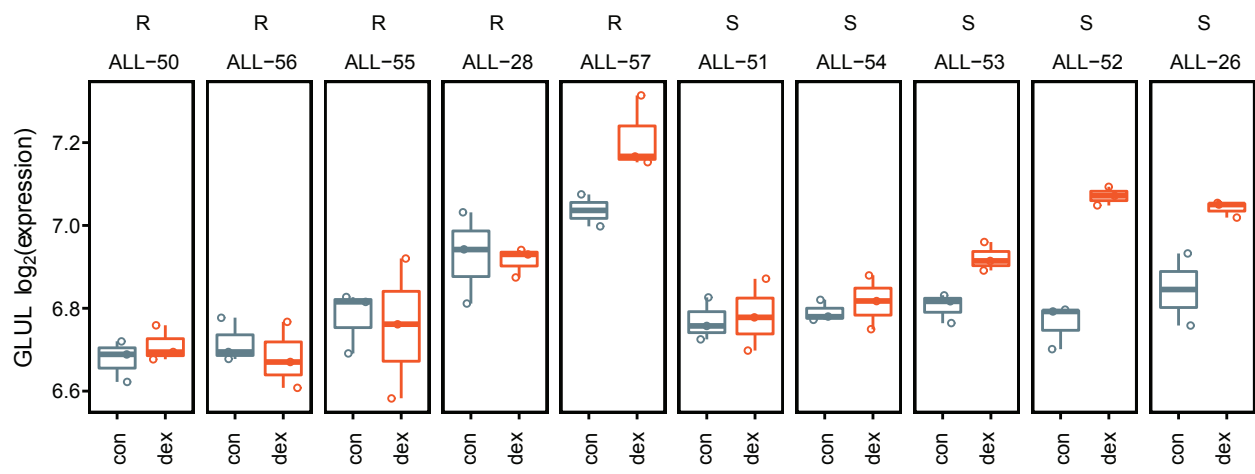

Supplement: Supplementary file 5 — Supplementary Figure 4 [file 41419_2018_625_MOESM5_ESM.pdf]

Supplementary Figure 5

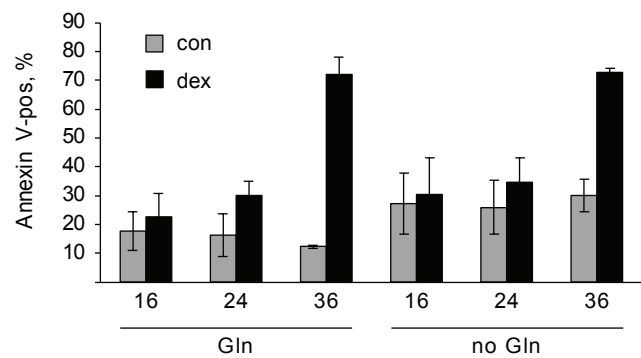

Supplement: Supplementary file 6 — Supplementary Figure 5 [file 41419_2018_625_MOESM6_ESM.pdf]
